# Predictive Factors of Cytomegalovirus Seropositivity among Pregnant Women in Paris, France

**DOI:** 10.1371/journal.pone.0089857

**Published:** 2014-02-24

**Authors:** Dieynaba S. N’Diaye, Yazdan Yazdanpanah, Anne Krivine, Thibaut Andrieu, Flore Rozenberg, Olivier Picone, Vassilis Tsatsaris, François Goffinet, Odile Launay

**Affiliations:** 1 Infection, Antimicrobiens, Modélisation, Evolution (IAME), Unité Mixte de Recherche (UMR) 1137, Institut National de la Santé et de la Recherche Médicale (INSERM), Université (Univ) Paris Diderot, Sorbonne Paris Cité, Paris, France; 2 Université Pierre et Marie Curie (UPMC) Univ Paris 06, École doctorale (ED)393, Paris, France; 3 Assistance-Publique Hôpitaux de Paris (AP-HP), Groupe Hospitalier Bichat-Claude Bernard, Hôpitaux Universitaires Paris Nord Val de Seine (HUPNVS), Service des Maladies Infectieuses, Université Paris Diderot, Sorbonne Paris Cité, Paris, France; 4 AP-HP, Groupe Hospitalier Cochin–Broca–Hôtel Dieu, Département de Virologie, Paris, France; 5 INSERM Unité (U)953, Recherche Epidémiologique en Santé Périnatale et Santé des Femmes et des Enfants, Université Pierre-et-Marie-Curie, Paris, France; 6 Université Paris Descartes, Paris Sorbonne Cité, Paris, France; 7 Hôpital Foch, Maternité, Suresnes, France; 8 AP-HP, Hôpital Cochin, Maternité, Paris, France; 9 INSERM Centre d’Investigation Clinique (CIC) P0901, Paris, France; 10 INSERM, CIC BT505, Paris, France; 11 AP-HP, Hôpital Cochin, CIC de Vaccinologie Cochin-Pasteur, Paris, France; University of Regensburg, Germany

## Abstract

**Background:**

Cytomegalovirus (CMV) is the most frequent cause of congenital infection. The objective of this study was to evaluate predictive factors for CMV seronegativity in a cohort of pregnant women in Paris, France.

**Methods:**

Pregnant women enrolled in a prospective cohort during the 2009 A/H1N1 pandemic were tested for CMV IgG antibodies. Variables collected were age, geographic origin, lifestyle, work characteristics, socioeconomic status, gravidity, parity and number of children at home. A multivariate logistic regression model was used to identify independent predictive factors for CMV seropositivity.

**Results:**

Among the 826 women enrolled, 389 (47.1%) were primiparous, and 552 (67.1%) had Metropolitan France as a geographic origin. Out of these, 355 (i.e. 57.0%, 95% confidence interval (CI): [53.6%–60.4%]) were CMV seropositive: 43.7% (95% CI:[39.5%–47.9%]) in those whose geographic origin was Metropolitan France and 84.1% in those with other origins (95% CI:[79.2%–88.3%]).

Determinants associated with CMV seropositivity in a multivariate logistic regression model were: (i) geographic origin (p<0.001(compared with Metropolitan France, geographic origins of Africa adjusted odds ratio (aOR) 21.2, 95% CI:[9.7–46.5], French overseas departments and territories and other origin, aOR 7.5, 95% CI:[3.9–14.6], and Europe or Asia, aOR 2.2, 95% CI: [1.3–3.7]); and (ii) gravidity (p = 0.019), (compared with gravidity = 1, if gravidity≥3, aOR = 1.5, 95% CI: [1.1–2.2]; if gravidity = 2, aOR = 1.0, 95% CI: [0.7–1.4]). Work characteristics and socioeconomic status were not independently associated with CMV seropositivity.

**Conclusions:**

In this cohort of pregnant women, a geographic origin of Metropolitan France and a low gravidity were predictive factors for CMV low seropositivity. Such women are therefore the likely target population for prevention of CMV infection during pregnancy in France.

## Introduction

Cytomegalovirus (CMV) is the most frequent cause of congenital infection in high-income countries. Approximately 1% of all newborns are infected by CMV at birth [1]. Of those infected, 10% are symptomatic and at high risk of developing permanent neurological or motor impairment, deafness, and blindness [2]–[5]. Among asymptomatic infected newborns, 5–10% will develop progressive hearing loss [2], [6], [7].

Primary and recurrent CMV infections have been observed during pregnancy [1], [3]. The risk of congenital infection is higher after maternal primary infection than after recurrent infection [1]. In France, as in most developed countries, around 50% of women of childbearing age are susceptible to CMV infection [8]–[11]. In CMV seronegative women, a 30% fetal transmission rate can be observed following primary infection during pregnancy [12].

Routine screening of women susceptible to CMV during pregnancy is controversial and not recommended in France, but the French National Institute for Public Health Surveillance (InVS) has estimated that 300,000 serodiagnostic tests are performed each year (2004 data), leading to costs and pregnancy-related stress (www.invs.sante.fr/publications/2007/cmv_grossesse). Routine screening is controversial because of scarce knowledge of the natural history of the disease, incomplete epidemiological data, and the fact that health interventions are limited and not consensual. It has, however, been stated that hygiene information on how to prevent CMV primary infection during pregnancy should be promoted, especially in CMV seronegative women [13]. Moreover, clinical trials on CMV vaccine candidates are promising, with several vaccine candidates at different stages of testing. In 2009, Pass et al reported promising results from a Phase II trial of one of these candidate vaccines demonstrating around 50% (95% CI: [7%–73%]) efficacy in preventing maternal primary infection [14]. With the potential arrival of new vaccines against CMV infection, there is an increased need to identify CMV seronegative non-pregnant women in order to prevent seroconversion during pregnancy. While the vaccine has yet not been tested on women with a pre-existing immunity, it is reasonable to believe that it could also help to prevent re-infection or reactivation. However, seropositive and seronegative women will probably not benefit from vaccination against CMV at the same extent since the risk of fetal transmission during pregnancy is reduced by the mother immunity [1]. Therefore the characterization of a target population of the vaccine could allow a more effective intervention.

Several studies have evaluated major determinants associated with seroprevalence, but none are recent enough to reflect current CMV epidemiology in France with a view to implementing an immunization campaign [10]. This study aims to characterize women susceptible to primary infection that would actually benefit from immunization campaign against CMV, and to assess in the French specific context, the predictive factors that would allow their identification.

## Materials and Methods

### Participants

The COFLUPREG “COhort on Flu during PREGnancy” study was a prospective cohort study conducted in pregnant women in three tertiary maternity centers in Paris (France) during the 2009 A/H1N1 influenza pandemic. 919 pregnant women randomly selected in order to obtain a representative sample of pregnant women followed up in these maternity hospitals were included from October 12, 2009 to February 3, 2010 to assess the incidence of serious forms of A/H1N1 influenza [15], [16]. Blood samples were obtained at inclusion in the cohort (between 6 and 35 weeks of gestation). Women>18 years old, understanding French were eligible to participate. Exclusion criteria were previous vaccination against the 2009 pandemic A/H1N1 influenza or virologically documented A/H1N1 influenza during the last 6 months. Sociodemographic (maternal age, geographic origin which includes ethnicity), lifestyle (single or couple) and obstetrical characteristics were collected at inclusion from medical records. Stored frozen serum from 826 of the women was available.

Some of the data collected were of interest for targeting pregnant women susceptible to CMV: sociodemographic characteristics, socioeconomic status (based on French National Institute for Statistics and Economic Studies (INSEE) nomenclatures for socioprofessional categories (http://www.insee.fr/nomenclatures), obstetrical characteristics (gravidity including the present pregnancy; parity excluding the present pregnancy) and factors associated with a higher risk of viral exposure and disease spreading (number of children<18 years old at home, work in contact with children and healthcare workers).

Written informed consent was obtained from each woman before enrolment. The protocol was conducted in accordance with the Declaration of Helsinki and French law for biomedical research and was approved by the “Ile-de-France 3” Ethics Committee (Paris, France). This study was not conducted outside of France and is registered with ClinicalTrials.gov: NCT01192737.

### Laboratory Methods of Titration to Determine CMV Seroprevalence

CMV IgG antibody levels were measured by ELISA using ETI-CYTOK-IgG Plus, Diasorin, Saluggia, Italy. CMV seropositivity was defined as a serum titer>400 IU/L.

### Statistical Methods

For each variable, the choice of the reference class was the one known to have the lowest CMV seropositivity rate and was made in accordance with the medical literature. When the literature lacked this information, the reference was the class with the highest number of women. Univariate analysis was done using the Chi2 test or Fisher’s exact test when class frequency was<5. The Cochran-Armitage test was used to test for trends.

Associations between determinants and CMV seroprevalence were analyzed using univariate analysis. Determinants with a p-value<0.20 on univariate analysis were included in the logistic regression model. A systematic search was made for interaction between determinants included. Then a backward elimination procedure and a model selection with the Akaike information criterion (AIC) was used to select the final multivariate model with factors associated significantly and independently with CMV seropositivity. Results were expressed as crude and adjusted odds ratios (aORs) with their 95% confidence interval (95% CI). Analyses were performed only for patients without missing data (complete case analysis) with SAS software version 9.3 (SAS Institute, Cary, NC).

## Results

### Study Population

Demographic profiles and clinical characteristics of the 826 women screened for CMV IgG are described in Table 1. Median age was 32.7 years (min: 18.8; max: 49.1), 47.1% of the women were primiparous, 67.1% had Metropolitan France as a geographic origin. The cohort had mainly a high (45.9%) or middle (40.2%) socioeconomic status; 6.7% of the women were single. The proportion of CMV seropositive women was 57.0% (95% CI: [53.6%–60.4%]). The proportion was 43.7% (95% CI: [39.5%–47.9%]) for women whose geographic origin was Metropolitan France, and 84.1% (95% CI: [79.2%–88.3%]) for those with other origins.

**Table 1:** Characteristics of the study population and determinants associated with seropositivity for CMV: univariate analysis.

Variables	Total	Seropositive for CMV	Crude OR	p-value †
	n = 826	n = 471	[95% CI]	
**Maternity hospital**				
Saint-Vincent de Paul	234	56.8%	1.2 [0.9–1.9]	
Port-Royal	396	60.1%	1.4 [1.0–2.0]	
Necker Brune	196	51.0%	1	0.001
**Age, years**				
15–29	190	64.2%	1	
30–34	322	52.8%	0.6 [0.4–0.9]	
35–39	207	53.6%	0.6 [0.4–1.0]	
≥40	107	63.5%	1.0 [0.6–1.6]	0.027
**Geographic origin** *				
Metropolitan France	552	43.7%	1	
European, Asian	66	62.1%	2.1 [1.3–3.6]	
Sub-Saharan African, North African	129	94.6%	22.5 [10.3–49.0]	
French overseas departments and territories, Other	76	85.5%	7.6 [3.9–14.8]	<0.001
**Socioprofessional category** **				
Low	114	73.7%	2.8 [1.8–4.5]	
Middle	332	59.3%	1.5 [1.1–2.0]	
High	379	49.9%	1	<0.001††
**Work at risk of CMV infection¥**				
No	389	55.0%	1	
Yes	437	58.8%	0.9 [0.7–1.1]	0.271
**Lifestyle** **				
Single	55	78.2%	2.9 [1.4–5.5]	
Couple	770	55.4%	1	0.001
**Gravidity**				
1	268	50.7%	1	
2	252	52.4%	1.1 [0.8–1.5]	
≥3	206	66.3%	1.9 [1.4–2.7]	<0.001††
**Parity**				
0	389	52.4%	1	
1	298	58.7%	1.3 [1.0–1.8]	
≥2	139	66.2%	1.8 [1.2–2.7]	0.015††
**Number of children <18 years old at home**				
0	399	52.9%	1	
1	290	60.0%	1.3 [1.0–1.8]	
≥2	137	62.8%	1.5 [1.0–2.2]	0.058††

† Chi 2.

†† Cochran-Armitage for trend with p<0.050.

¥ Work at risk included health care workers and job in contact with children.

*3 missing values.

**1 missing value.

### Univariate Analysis

Sociodemographic and obstetrical determinants significantly associated with CMV seropositivity were maternal age (p = 0.027), geographic origin (p<0.001), lifestyle (p = 0.001), socioprofessional category (p<0.001), gravidity (p<0.001), and parity (p = 0.015) (Table 1). No significant association was found between CMV seropositivity and job characteristics (p = 0.271) or number of children<18 years old at home (p = 0.058), although this factor was borderline significant.

Seropositivity for CMV decrease with socioeconomic status and increased when gravidity, parity, or number of children<18 years old at home increased (p<0.050). CMV seropositivity rate was the lowest for women whose geographic origin was Metropolitan France than for those of Europe or Asia; the highest proportion was observed for women with Sub-Saharan and North Africa origins (p<0.001). The seropositivity rate was higher in younger women (64.2%) and decrease for women in the 30–34 and 35–39 age groups (52.8% and 53.6%, respectively). However, women≥40 had a seropositivity of 63.5%, so no linear trend was observed between age and seropositivity.

### Multivariate Logistic Regression

Determinants independently associated with CMV seropositivity were geographic origin (p<0.001) (compared with Metropolitan France, geographic origins of Africa adjusted odds ratio (aOR) 21.2, 95% CI:[9.7–46.5], French overseas departments and territories and other origin, aOR 7.5, 95% CI:[3.9–14.6], and Europe or Asia, aOR 2.2, 95% CI:[1.3–3.7]); and gravidity (p = 0.019) (compared with gravidity = 1, if gravidity≥3, aOR = 1.5, 95% CI:[1.1–2.2]; if gravidity = 2, aOR = 1.0, 95% CI:[0.7–1.4]) (Table 2).

**Table 2:** Determinant associated with seropositivity for CMV: multivariate analysis including all determinants with a p-value<0.20 in the univariate analysis.

Variables	Adjusted OR	p-value†
	[95% CI]	
**Geographic origin** *		
Metropolitan France	1	
European, Asian	2.2 [1.3–3.7]	
Sub-Saharan African, North African	21.2 [9.7–46.5]	
French overseas departments and territories, Other	7.5 [3.9–14.6]	<0.001
**Gravidity**		
1	1	
2	1.0 [0.7–1.4]	
≥3	1.5 [1.1–2.2]	0.019

CI: Confidence Interval.

† Chi 2.

Variables with a p-value<0.20 in univariate analysis were included in the multivariate analysis.

Final logistic regression model was obtained with a backward elimination and with comparison of the Akaike Information Criterion (AIC).

*3 missing values.

## Discussion

Data from the COFLUPREG prospective cohort allow us to study numerous determinants associated with CMV seropositivity and their relative importance. The study was multicentric involving three major university maternity hospitals in Paris in 2009 and a large number of blood samples recently and randomly collected was available (n = 826). This study design and the quality of the data reinforce the reliability of the results.

To our knowledge, in France, or in other West European countries, no other recent results on the characterization of women susceptible to CMV infection that highlight, like in this study, the discrepancy of seroprevalence between women of African origin and those of French origin. Paris Metropolitan area, even if not representative of all French population, is an area with a high proportion of immigrants from sub-Saharan Africa and their children. Consequently, this study allowed us to specifically explore CMV in this population. Even if seroprevalence point estimates from this study cannot be extrapolated to other regions in France, they are valid and indicative of the fact that CMV epidemiology is very different among women of African origin living in France compared to other sub-groups and the overall population.

These results may be valuable when candidate vaccines against CMV are tested to identify the population for which vaccination should be recommended.

However, this study also presents several limitations. First, the primary objective of the cohort was not linked to study of CMV infection. Therefore, several determinants associated with CMV seropositivity were not collected. This was the case, for example, of sexual behavior, although sexual transmission of CMV from a sexual partner is well known to be an infection route [17]. It could also be the reason why the number of children was not found to be associated with CMV seropositivity since studies have shown that mothers are mainly infected after contact with very young children (more likely under 36 months), whereas in Coflupreg the variable included also women with older children [18].

Second, our study population comes from three university maternity hospitals in Paris; it is therefore not representative of all French pregnant women. Thus, compared with the general population of Metropolitan France in 2009, maternal age during pregnancy of our study population was higher (32.7 vs. 29.9), as was the proportion of primiparous women (47.1% vs. 44%), and high socioeconomic status was more represented at the expense of middle socioeconomic status (INSEE 2009 http://www.insee.fr/fr/Données statistiques naissances 2009).

All the pregnant women enrolled came from an urban area which might have had an impact on seropositivity rate since it has been shown that women from a rural area tend to have a higher seropositivity rate [18]. Gratacap et al have shown that place of birth also has an impact on CMV seroprevalence, with a North-South gradient even within France, with women born in the southern regions having a higher seropositivity rate [10], [19].

Consequently, results such as the CMV seropositivity and distribution of sociodemographic factors cannot be extrapolated to all French pregnant women. But, this limitation probably does not interfere with the analysis of associations between studied determinants and CMV seropositivity among pregnant women.

In this cohort of French pregnant women conducted during the 2009 A/H1N1 pandemic, seropositivity for CMV was 57.1%. Metropolitan France as a geographic origin and a low gravidity were the predictive factors for low CMV seropositivity. Seropositivity was slightly lower than the rate reported in previous French studies on pregnant women: 50.1% in the 2009 study by Vauloup-Fellous et al [9], 44.6% in the 2000 study by Gouarin et al [11] and 51.5% in the 1998 study by Gratacap-Cavallier et al [10]. This could be due to the high proportion of women of African origin (16% vs. 6% in the Gratacap-Cavallier et al study, for example). Pregnant women whose geographic origin was Metropolitan France had a seropositivity rate of 43%, which was close to the range (41% to 59%) reported in other high income countries [20]–[24].

In the present study, seropositivity rate significantly increased with increase in parity, gravidity, number of children<18 years old at home, and with decrease in socioprofessional category. It was also strongly associated with place of birth, with a North-South gradient with a lower seropositivity rate in the North. Having Metropolitan France as a geographic origin, having a higher socioeconomic status and no children were predictive factors of lower seropositivity rates. These risk factors confirm those described in the literature. Major disparities in seroprevalence rates according to geographic origin could be related to differences in breastfeeding frequency and duration [25], hygiene level during childhood, propensity to have children that attend day-care centers and sexual behavior [17]. Geographic origin was recorded using medical records taken during pregnancy and was primarily collected in order to assess the risk of ethnicity-related diseases. Consequently, a woman, for example, of African origin could be born in France to African parents or in Africa. It is therefore difficult to assess if high seropositivity in women of African origin is due to place of birth or to ethnicity.

The association between high socioeconomic status and low seropositivity rate may be due to more hygienic living conditions in less crowded households with less exposure to young children due to birth control. Moreover, since a frequently described key risk factor for seroconversion is being a parent of a child who is shedding CMV, the tendency for primiparous women to not be seropositive is not surprising.

Unlike in other studies, no linear relationship between age and seropositivity for CMV was found [10], [26]. This was attributable to the fact that pregnant adolescent and young women in the age group 15–29 years had a much higher rate of CMV seropositivity than other age groups. Further analysis of this age group showed that those women had a significantly lower socioeconomic status and were less likely than the rest of the cohort to have Metropolitan France as a geographic origin, which could explain their high seroprevalence (data not shown). Fowler et al reported higher seroconversion rates n during pregnancy for women≤25 years compared with the general population [17].

These results clearly show that not every woman in France is at the same risk of CMV infection. Indeed, it is very likely that if women with certain predictive factors are seropositive for CMV at 95%, a vaccine preventing recurrent infections may not be cost-effective, because it will probably cost a lot for a little improvement of health in that population. In a context where the French High Authority for Health requires a cost-effectiveness analysis when a new health product shows an improvement in the medical benefit and is likely to have a significant impact on the social security’s health expenditure, (Décret n° 2012–1116 du 2 octobre 2012 relatif aux missions médico-économiques de la Haute Autorité de santé), a universal immunization, for example, at the beginning of the reproductive lives of women, as for HPV vaccine, might not be the most efficient measure to fight CMV infections in all French diversified sub-populations. Use of serological screening and predictive factors such as Metropolitan France origin and low gravidity, to identify susceptible women might be less costly and should be further explored in a comprehensive cost-effectiveness analysis. Money saved could be reallocated to other types of primary prevention that have been shown to actually improve clinical outcomes like education for health of both women and health care workers, and hygiene counseling [27], [28].

In 2010, 81.7% of 820,000 births were to mothers born in Metropolitan France, and approximately 80% were their first or second born (INSEE, 2010 http://www.insee.fr/Statistiques des naissances 2010). This is probably the population that should be primarily targeted for vaccine use. However, since prevalence of CMV infection is tending to decrease, women not primarily targeted for anti-CMV vaccination might have a decreasing seroprevalence in the future and be in need of immunity [18], [29], [30].

However, our results should probably be confirmed by a national sero-epidemiological study with results on both seroprevalence and predictive factors of the CMV infection. And, further research is needed to estimate, based on effectiveness and cost-effectiveness outcomes, the threshold of the global seroprevalence at which routine vaccination for every woman in France would become an effective strategy.

## Conclusions

In conclusion, in a large prospective study conducted in pregnant women, the proportion of pregnant women susceptible to CMV seroconversion was high among those whose geographic origin was Metropolitan France and whose gravidity was low. This is the population that should be targeted for CMV prevention in general and for a potential CMV vaccination strategy.
